# Supplementary material for: The Control of Postharvest Soft Rot Caused by Rhizopus stolonifer on Kokei No. 14 Organic Sweet Potato Roots by Carvacrol, Thymol, and Thyme Oil
Source: Foods. 2025 Apr 5;14(7):1273. doi: 10.3390/foods14071273 (PMC11989222; doi:10.3390/foods14071273)
Supplement: Supplementary file 1 [file foods-14-01273-s001.zip › foods-3516602-supplementary.pdf]

Table S1. Minimum inhibitory concentrations of selected essential oils against mycelial growth of *Rhizopus stolonifer* on potato dextrose agar.

Common name	Botanical name or chemical formula	Minimum inhibitory concentration (mg/L)		Source
		R. stolonifer		
		Contact	Vapor	
Pure component essential oils				
Thymol	$C_{10}H_{14}O$	100	10	Sigma, USA
Carvacrol	$C_{10}H_{14}O$	200	10	Sigma, USA
Eugenol	$C_{10}H_{12}O_2$	500	100	Sigma, USA
Citral	$C_{10}H_{16}O$	>500	50	Sigma, USA
Trans-cinamaldehyde	C_9H_8O	200	50	Yuanye, China
Vanilla	$C_8H_8O_3$	>500	100	Yuanye, China
L-carvone	$C_{10}H_{14}O$	200	50	Sigma, USA
Crude extract of essential oils				
Thyme oil	*Thymus vulgaris*	100	10	Sigma, USA
Oregano oil	*Origanum vulgare*	>500	50	Yuanye, China
Lemongrass oil	*Cymbopogon citratus*	>500	100	Sigma, USA
Spearmint oil	*Mentha spicata*	>500	200	Sigma, USA

Table S2 List of detail properties of each sensor used in the electronic nose (E-nose) system.

Sensor Numbers	Sensor Name	Main Response Compound Types	Volatile Compound Objects
Sensor1	SN-1	Alkanes, smoke	C_3H_8, CH_4, smoke
Sensor2	SN-2	Alcohols, aldehydes, short-chain alkanes	C_2H_6O, C_4H_{10}, CH_2O, smoke
Sensor3	SN-3	Ozone	O_3
Sensor4	SN-4	Sulfides	H_2S
Sensor5	SN-5	Organic Amines	NH_3, CH_3NH_2, C_2H_7NO
Sensor6	SN-6	Organic gases, benzene, ketones, alcohols, aldehydes, aromatic compounds	$C_6H_5CH_3$, C_3H_6O, C_2H_6O, H_2, other organic gases
Sensor7	SN-7	Short-chain alkanes	CH_4
Sensor8	SN-8	Short-chain alkanes	C_3H_8, liquefied gases
Sensor9	SN-9	Aromatic, alcohols and aldehydes compounds	$C_6H_5CH_3$, CH_2O, C_6H_6, C_2H_6O, C_3H_6O
Sensor10	SN-10	Hydrogenated gases	H_2
Sensor11	SN-11	Alkanes and olefins compounds	C_nH_{2n+2}, C_nH_{2n}, liquefied gases
Sensor12	SN-12	Short-chain alkanes	CH_4, liquefied gases
Sensor13	SN-13	Combustible gases	CH_4
Sensor14	SN-14	Combustible gases	Combustible gases, smoke
Sensor15	SN-15	Alkanes, organic gases	C_4H_{10}, organic acid ester, aliphatic hydrocarbon, smoke
Sensor16	SN-16	Sulfides	Sulfurous
Sensor17	SN-17	Nitrides	Nitrogen oxides
Sensor18	SN-18	Ketones and alcohols	C_3H_6O, C_2H_6O, organic solvents

Table S3 List of sensory descriptive attributes of sweet potato roots treatments.

Number	Attributes	0-3			4-7				8-10		
1	Appearance	Poor appearance			General appearance				Good appearance		
		1	2	3	4	5	6	7	8	9	10
2	Flesh color	Poor color			General color				Good color		
		1	2	3	4	5	6	7	8	9	10
3	Firmness	Not hard			Moderate hard				Very hard		
		1	2	3	4	5	6	7	8	9	10
4	Sweet potato odor	No odor			light odor				Strong odor		
		1	2	3	4	5	6	7	8	9	10
5	Off-odor	No off-odor			Light off-odor				Strong off-odor		
		1	2	3	4	5	6	7	8	9	10
6	Sweetness	Less sweet			Moderate sweet				Very sweet		
		1	2	3	4	5	6	7	8	9	10
7	Fibrousness	Less fibrous			Moderate fibrous				Very fibrous		
		1	2	3	4	5	6	7	8	9	10
8	Viscosity	Less viscosity			Moderate viscosity				Very viscosity		
		1	2	3	4	5	6	7	8	9	10

9	Off-flavor	No off-flavor			Light off-flavor				Strong off-flavor		
		1	2	3	4	5	6	7	8	9	10
10	Overall assessment	Unacceptable			Barely acceptable				Acceptable		
		1	2	3	4	5	6	7	8	9	10

Table S4 Analysis of Variance (ANOVA) of Mean Sensory Scores for Essential Oil Treated Sweet Potato Roots.

Treatments			Appearance (AR)	Flesh color (FC)	Firmness (FM)	Sweet potato odor (SO)	Off-odor (OO)	Sweetness (SW)	Fibrousness (FB)	Viscosity (VS)	Off-flavor (OF)	Overall assessment (OA)
7d	Control		8.20±0.84^{a}	7.40±0.55^{b}	5.20±0.45^{a}	8.40±0.89^{a}	2.00±0.71^{g}	6.60±0.55^{a}	3.40±0.55^{a}	6.80±0.84^{a}	2.00±0.71^{e}	9.00±0.32^{a}
	Carvacro	100mg/L	8.40±0.55^{a}	8.20±0.84^{ab}	4.80±0.84^{a}	7.20±0.84^{bcd}	4.40±0.89^{d}	6.80±0.84^{a}	2.80±0.45^{ab}	7.20±0.84^{a}	4.80±0.84^{c}	7.40±0.40^{c}
		300mg/L	8.40±1.14^{a}	8.20±0.45^{ab}	5.20±0.84^{a}	7.40±0.89^{abc}	4.60±0.55^{d}	6.80±1.10^{a}	2.80±0.84^{ab}	7.20±0.84^{a}	5.20±0.84^{c}	7.00±0.32^{cd}
		500mg/L	8.60±0.55^{a}	8.40±0.55^{ab}	5.20±0.84^{a}	6.40±0.55^{cd}	5.60±0.55^{c}	6.80±0.45^{a}	2.80±0.84^{ab}	7.20±0.45^{a}	6.20±0.45^{b}	6.20±0.37^{d}
	Thymol	100mg/L	8.40±0.55^{a}	8.20±0.84^{ab}	5.40±0.55^{a}	7.60±0.89^{ab}	2.20±0.45^{g}	6.80±1.30^{a}	2.80±1.10^{ab}	7.20±0.84^{a}	2.80±0.84^{de}	8.60±0.24^{ab}
		300mg/L	8.60±0.89^{a}	8.40±0.55^{ab}	5.60±0.55^{a}	7.40±0.55^{abc}	2.80±0.45^{fg}	7.20±0.45^{a}	2.00±0.71^{b}	7.40±0.89^{a}	3.20±0.84^{d}	7.60±0.24^{bc}
		500mg/L	8.60±0.55^{a}	8.40±0.89^{ab}	5.60±0.89^{a}	7.00±1.00^{bcd}	3.80±0.45^{de}	7.20±0.84^{a}	2.00±1.00^{b}	7.40±0.55^{a}	3.60±0.55^{d}	7.00±0.45^{cd}
	Thyme oil	100mg/L	8.60±1.14^{a}	8.40±0.55^{ab}	5.80±0.84^{a}	6.40±0.55^{cd}	3.20±0.45^{ef}	7.40±0.89^{a}	2.00±0.71^{b}	7.40±1.14^{a}	3.80±0.84^{d}	8.00±0.32^{abc}
		300mg/L	8.60±0.89^{a}	8.40±0.89^{ab}	5.80±1.10^{a}	6.20±0.45^{d}	6.40±0.55^{b}	7.20±0.84^{a}	2.00±0.71^{b}	7.40±0.55^{a}	6.20±0.84^{b}	6.20±0.37^{d}
		500mg/L	8.60±0.55^{a}	8.60±0.55^{a}	5.80±0.84^{a}	6.20±0.84^{d}	8.20±0.84^{a}	7.20±1.10^{a}	2.00±1.00^{b}	7.40±0.89^{a}	7.40±0.55^{a}	4.60±0.40^{e}

	Control		7.80 ± 0.84^{a}	7.20 ± 0.84^{a}	4.80 ± 0.84^{a}	8.40 ± 0.89^{a}	2.00 ± 0.71^{c}	6.80 ± 0.84^{a}	3.80 ± 0.84^{a}	7.20 ± 0.84^{a}	2.00 ± 0.71^{d}	9.00 ± 0.71^{a}
	Carvacrol	100mg/L	8.20 ± 0.84^{a}	8.00 ± 0.71^{a}	4.80 ± 0.45^{a}	7.60 ± 0.89^{ab}	2.20 ± 0.84^{bc}	7.20 ± 0.84^{a}	3.40 ± 0.55^{ab}	7.40 ± 0.55^{a}	2.20 ± 0.45^{cd}	8.60 ± 0.89^{ab}
		300mg/L	8.20 ± 0.45^{a}	8.00 ± 1.00^{a}	5.20 ± 0.45^{a}	7.60 ± 0.55^{ab}	2.80 ± 0.84^{bc}	7.20 ± 0.45^{a}	3.00 ± 1.00^{ab}	7.40 ± 0.89^{a}	2.80 ± 0.84^{bcd}	8.40 ± 0.55^{ab}
		500mg/L	8.40 ± 0.89^{a}	8.20 ± 0.84^{a}	5.20 ± 0.84^{a}	7.40 ± 0.55^{ab}	3.20 ± 0.84^{b}	7.20 ± 0.84^{a}	3.00 ± 0.71^{ab}	7.40 ± 0.55^{a}	3.60 ± 0.89^{b}	7.60 ± 0.89^{bc}
14 d	Thymol	100mg/L	8.20 ± 0.84^{a}	8.00 ± 0.71^{a}	5.20 ± 1.10^{a}	7.60 ± 0.89^{ab}	2.20 ± 0.45^{bc}	7.00 ± 1.00^{a}	3.00 ± 0.71^{ab}	7.40 ± 0.89^{a}	2.20 ± 0.84^{cd}	8.60 ± 0.89^{ab}
		300mg/L	8.40 ± 0.55^{a}	8.20 ± 0.45^{a}	5.40 ± 0.89^{a}	7.40 ± 0.55^{ab}	2.20 ± 0.84^{bc}	7.20 ± 0.84^{a}	2.40 ± 0.55^{b}	7.40 ± 0.55^{a}	2.80 ± 0.84^{bcd}	8.40 ± 0.55^{ab}
		500mg/L	8.40 ± 0.89^{a}	8.20 ± 0.84^{a}	5.40 ± 0.55^{a}	7.40 ± 1.14^{ab}	3.20 ± 0.84^{b}	7.20 ± 0.45^{a}	2.40 ± 0.55^{b}	7.40 ± 0.89^{a}	3.20 ± 0.84^{bc}	8.00 ± 0.71^{abc}
	Thyme oil	100mg/L	8.40 ± 0.55^{a}	8.20 ± 1.10^{a}	5.60 ± 0.89^{a}	7.20 ± 0.84^{ab}	2.80 ± 0.84^{bc}	7.60 ± 0.89^{a}	2.40 ± 0.89^{b}	7.40 ± 0.55^{a}	2.80 ± 0.84^{bcd}	8.60 ± 0.55^{ab}
		300mg/L	8.40 ± 0.89^{a}	8.20 ± 0.45^{a}	5.60 ± 0.55^{a}	7.00 ± 1.00^{b}	3.20 ± 0.84^{b}	7.40 ± 0.55^{a}	2.40 ± 0.55^{b}	7.40 ± 0.55^{a}	3.60 ± 0.89^{b}	8.00 ± 0.71^{abc}
		500mg/L	8.40 ± 0.55^{a}	8.20 ± 1.10^{a}	5.80 ± 0.84^{a}	6.80 ± 0.84^{b}	4.60 ± 0.89^{a}	7.40 ± 0.89^{a}	2.40 ± 0.89^{b}	7.40 ± 0.89^{a}	4.80 ± 0.84^{a}	7.00 ± 0.71^{c}
